# Eucalyptol

**Published:** 1889-11

**Authors:** 


EUCALYPTOL.—We learn that the firm of Sander & Son, of Australia, are the only firm which keep extensive works for the manufacture of their preparation, *the Pure, valuable Eucalyptol Extract.* (Eucalyptol). According to Prof. Hugo Schuly, mature three year old leaves have to be worked in their green state to secure a genuine article. To accomplish this, factories have been erected on the Australian continent by this firm, which may be relied upon as preparing a genuine article.

THE N. Y. MEDICAL RECORD should have been credited with the article entitled, "A successful case of intubation," in the Oct. No. of our Journal.

